# Identification of the recently described plasminogen gene mutation p.Lys330Glu in a family from Northern Germany with hereditary angioedema

**DOI:** 10.1186/s13601-019-0247-x

**Published:** 2019-02-14

**Authors:** Andreas Recke, Elisabeth G. Massalme, Uta Jappe, Lars Steinmüller-Magin, Julia Schmidt, Yorck Hellenbroich, Irina Hüning, Gabriele Gillessen-Kaesbach, Detlef Zillikens, Karin Hartmann

**Affiliations:** 1 Department of Dermatology, Allergology and Venereology, University of Lübeck, Ratzeburger Allee 160, 23538 Lübeck, Germany; 2 Division of Clinical and Molecular Allergology, Priority Research Area Asthma and Allergy, Research Center Borstel, Borstel (Sülfeld), Germany; 3 Airway Research Center North (ARCN), Member of the German Center for Lung Research (DZL), Borstel (Sülfeld), Germany; 4 Interdisciplinary Allergy Outpatient Clinic, Department of Pneumology, University of Lübeck, Lübeck, Germany; 5 Institute of Laboratory Medicine and Human Genetics, Singen, Germany; 6 Institute of Human Genetics, University of Göttingen, Göttingen, Germany; 7 Institute of Human Genetics, University of Lübeck, 23538 Lübeck, Germany

## Abstract

Hereditary angioedema (HAE) is a life-threatening disease characterized by recurrent episodes of subcutaneous and mucosal swellings and abdominal cramping. Corticosteroids and antihistamines, which are usually beneficial in histamine-induced acquired angioedema, are not effective in HAE. Therefore, diagnosing HAE correctly is crucial for affected patients. We report a family from Northern Germany with six individuals suffering from recurrent swellings, indicating HAE. Laboratory tests and genetic diagnostics of the genes *SERPING1*, encoding C1 esterase inhibitor (C1-INH), and *F12,* encoding coagulation factor XII, were unremarkable. In three affected and one yet unaffected member of the family, we were then able to identify the c.988A > G (also termed c.1100A > G) mutation in the *plasminogen* (*PLG)* gene, which has recently been described in several families with HAE. This mutation leads to a missense mutation with an amino acid exchange p.Lys330Glu in the kringle 3 domain of plasminogen. There was no direct relationship between the earlier described cases with this mutation and the family we report here. In all affected members of the family, the symptoms manifested in adulthood, with swellings of the face, tongue and larynx, including a fatal case of a 19 year-old female individual. The frequency of the attacks was variable, ranging between once per year to once a month. In one individual, we also found decreased serum levels of plasminogen as well as coagulation factor XII. As previously reported in patients with PLG defects, icatibant proved to be very effective in controlling acute attacks, indicating an involvement of bradykinin in the pathogenesis.

## To the editor

Hereditary angioedema (HAE) is a life-threatening disease associated with recurrent episodes of subcutaneous and mucosal swellings and painful abdominal cramping [1]. Anti-allergic drugs, i.e. antihistamines, corticosteroids and epinephrine, which are administered in histamine-mediated angioedema, are not effective in the treatment of HAE. Therefore, a correct and early diagnosis is of utmost importance for affected individuals in order to transfer them to specialized medical centers and to provide effective emergency medications. We here report a family with HAE from Northern Germany, in which we were able to identify the recently described c.988A > G (p.Lys330Glu) mutation in the *plasminogen* (*PLG)* gene.

**Fig. 1:**
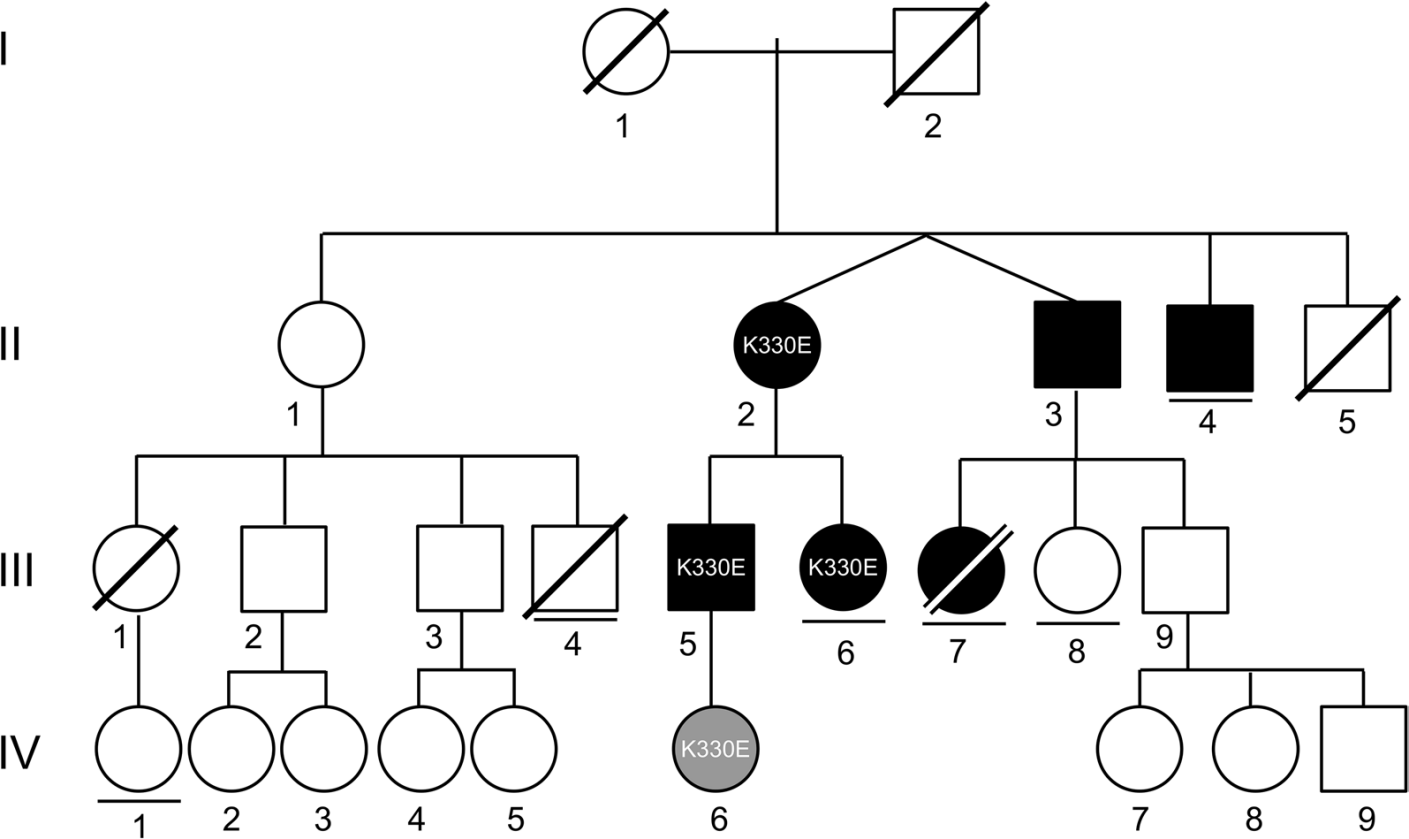
Pedigree of a family with hereditary angioedema associated with a *plasminogen* mutation (HAE-PLG). Symbol description: Circles indicate females, squares males. Black filled symbols indicate affected individuals, gray filled symbols mutation carriers without symptoms. A slash indicates a deceased individual. A horizontal line below a symbol indicates that the (adult) individual has no children. Roman numerals indicate generations, Arabic numerals individuals within a generation. “K330E” in circles or squares indicates individuals who were available for genotyping and showed the *plasminogen* mutation p.Lys330Glu mutation (K330E). The other affected individuals were so far not available for genotyping

The female index patient (Fig. 1; patient II-2) aged 69 years presented in 2014 with recurrent swellings of tongue and lips, and sometimes also larynx, throat and upper airways. Swelling attacks occurred 1–4 times yearly. Additionally, she suffered from abdominal cramping 2–3 times yearly. The symptoms had started in 2006 at the age of 61 years. Initially, we excluded allergic causes of the recurrent swellings by history and extensive in vivo and in vitro allergy diagnostic testing (skin prick testing and IgE detection). complement factor C4 and C1 esterase inhibitor (C1-INH) concentration as well as function were also unremarkable. The clue for the diagnosis of HAE was then provided by a renewed report of the patient addressing her family history. She reported that her twin brother (patient II-3) has similar symptoms, and his daughter (patient III-7) had died from suffocation due to laryngeal edema at the age of 19 years. Moreover, her two children (patients III-5 and III-6) suffered from similar symptoms. In all cases, the index patient treated swelling attacks immediately with the bradykinin B-2 receptor inhibitor icatibant 30 mg subcutaneously, and swellings responded within 15–20 min after treatment [1, 2]. The son of the index patient (patient III-5), aged 42 years, developed swellings of the lips after he had started an angiotensin-converting enzyme (ACE) inhibitor for treatment of hypertension. To our knowledge, patient III-5 is the only one of this family who has received an ACE inhibitor. After discontinuation of this treatment, he remained free of symptoms. This patient has a 12 year-old daughter (patient IV-6), who did not experience any symptoms until now, although she was later on also identified as a carrier of the p.Lys330Glu mutation.

The daughter of the index patient (patient III-6), aged 37 years, reported recurrent swellings of the lips and tongue. Compared to her mother, the frequency of attacks was lower, about 1–2 per year. Rarely, these swellings were also accompanied by abdominal pain. The attacks had started at the age of 20 years. Until now, she never developed a more severe attack with laryngeal edema or dyspnea. For the treatment of acute attacks, icatibant was found to be as effective as in her mother (patient II-2).

In the index patient (patient II-2), genetic analyses for mutations in the *SERPING1* (C1-INH) and *F12* (coagulation factor XII) genes were negative. For more then 3 years, the diagnosis of HAE was therefore solely based on the family history and the response to treatment with icatibant off-label. At that time, it was not possible to verify the diagnosis, until the first report of a mutation in the *PLG* gene became available [2].

Using Sanger sequencing of exon 9 of the *PLG* gene, we then identified the previously described NM_000301.3:c.988A > G (NP_000292.1:p.Lys330Glu) *PLG* mutation (also termed c.1100A > G; p.Lys311Glu) in four family members (patients II-2, III-5 and III-6 and IV-6). Based on these findings, we were now able to offer accurate genetic counselling and, importantly, predictive genetic analysis for so far unaffected members.

Comparing our family with the recently published patients with HAE associated with a *PLG* gene mutation (HAE-PLG) [2–6], the age of onset, clinical presentation and frequency of angioedema of all affected members of our family are in line with these previous reports. Patients with HAE-PLG apparently tend to develop first clinical symptoms in adulthood, whereas patients with defects in C1-INH (HAE-C1-INH) usually start with their symptoms during childhood. The clinical picture of recurrent tongue swellings can be considered as indicative for HAE with normal C1-INH (HAE-nC1-INH). Abdominal attacks may also occur, but are less frequent than in HAE-C1-INH [7, 8].

With our family, there are now 24 unrelated families from different countries with HAE-PLG with 105 affected individuals (Table 1) [2, 3]. The number of 89 affected individuals of German origin corresponds to a prevalence of about 1 patient per million inhabitants in Germany, although the number of unrecorded cases must be expected to be higher. In all individuals, the same point mutation in the *PLG* gene was found, although they were reported to be unrelated. A founder effect was demonstrated in 4 of the families with the PLG gene defect [2]. ACE inhibitors and AT1R inhibitors were reported to induce acute attacks—and sometimes even the first attack—in patients with PLG gene defects [4–6], such as in our patient III-6. Therefore, consideration should be given to whether a PLG defect should be investigated in all cases of ACE inhibitor-induced angioedema.

**Table 1:** Overview of reported cases with HAE-nC1-INH and the plasminogen mutation PLG:p.Lys330Glu mutation

Reported families (N) and ethnic origin	Males (N)/females (N)	Mean age of onset (years)	Fatal cases	Treatment	References
13 German	13/47	30.5	2 female cases	ICA, TXA	Bork et al. [2]
3 German	7/15	*n.a.*	*n.a.*	*n.a.*	Dewald [3]
1 Greece	3/1	*n.a.*	*1 male case*	*n.a.*	Germenis et al. [11]
1 Bulgarian					
2 Spanish^a^					
3 French	2/6	*6*-*64*	*none*	TXA	Belbézier et al. [12]
2 Japanese	1/3	26-94	none	TXA	Yakushiji H et al. [13]
1 German	3/4	~ 20-60	1 female case	ICA	*Present report*

All patients shown in this table had the PLG:p.Lys330Glu mutation. Dewald [3] uses a different nomenclature (NM_000301.3:c.1100A > G, p.Lys311Glu) for this mutation. However, the two seemingly different locations correspond to the same amino acid residue A_330_ (in Uniprot entry P00747-1, GenBank entry NP_000292.1). A_311_ is the amino acid residue after subtraction of the 19 amino acids long signal peptide (amino acid residues M_1_-G_19_). Nucleotide position 1100 and 988 refer to the same position in the sequence of plasminogen transcript 1 (in NBCI RefSeq NM_000301.3). However, 1100 is the position relative to the first nucleotide in the RefSeq entry, while 988 is the position relative to the start codon (nucleotide 113). To follow the more commonly used nomenclature and to adhere to the amino acid and nucleotide positions relative to the coding sequence start as listed in the NCBI database entries, we chose the designation NM_000301.3:c.988A > G (NP_000292.1:p.Lys330Glu)

*ICA* icatibant, *TXA* tranexamic acid

^a^Both cases carried an additional c.266G > A, p.Arg89Lys mutation in the PLG gene [6]

While treatment guidelines exist for HAE-C1-INH types I and II, there is insufficient evidence to recommend a specific therapy or management strategy for HAE-nC1-INH [1, 2, 7]. Clinical evidence suggests that bradykinin may play a major role in some types of HAE-nC1-INH [1, 2, 9]. In accordance, in our family, icatibant was found to be effective for the treatment of acute attacks. A response to long-term treatment with tranexamic acid 3 g/d was described by Bork et al. [2]. Controlled clinical trials, however, are needed to investigate whether treatment options used for HAE-C1-INH, such as BCX7353, avoralstat, ecallantide, conestat alfa, purified C1-INH and a recombinant antibody inhibiting kallikrein (lanadelumab), are also effective in HAE-nC1-INH [1, 7, 10–13].

Based upon the recent reports of mutations in the *PLG* and angiopoietin-1 (*ANGPT1*) genes associated with HAE [2, 3, 9], in addition to the previously known *SERPING1* and *F12* mutations, a novel classification of HAE was proposed [14]. In general, HAE is here divided into two major groups, HAE-C1-INH and HAE-nC1-INH. HAE-C1-INH is subdivided into (1) reduced plasma concentration of C1-INH and (2) dysfunctional C1-INH with normal plasma concentration. Both variants can be easily detected by measuring C1-INH protein concentration and activity as well as C4. Further, HAE-nC1-INH is now subcategorized into (1) HAE-FXII, (2) HAE-ANGPT1, (3) HAE-PLG and (4) HAE-unknown, according to the respective mutational profile [14]. Identification of patients with HAE-nC1-INH and exclusion of possible differential diagnoses is often challenging and requires careful work-up. It is important to note that gene defects should also be excluded in seemingly unaffected family members, because the swellings can start at nearly any age [2]. The diagnostic workup of angioedema should exclude the possibility of acquired angioedema, i.e. ACE-inhibitor associated angioedema (ACEI-AAE), acquired C1-deficiency angioedema (C1-INH-AAE) and acquired non-histaminergic angioedema (InH-AAE) [15].

HAE-nC1-INH is a life-threatening disease that is, lacking appropriate biomarkers, difficult to diagnose. Therefore, it is of importance to sensitize physicians for this rare and severe, but treatable disease which might be concealed as an ACE inhibitor-induced angioedema or acquired non-histaminergic angioedema.

## Abbreviations

HAE: hereditary angioedema
PLG: plasminogen gene
ANGPT1: angiopoietin 1 gene
SERPING1: C1-INH gene
F12: coagulation factor XII gene
C1-INH: complement factor 1 inhibitor
HAE-nC1-INH: HAE with normal C1-INH levels or function
HAE-C1-INH: HAE with with deficient C1-INH
HAE-PLG: HAE with PLG gene defect
HAE-ANGPT1: HAE with ANGPT1 gene defect
HAE-FXII: HAE with F12 gene defect
